# Editorial: TCM approaches in cellular endocrinology

**DOI:** 10.3389/fendo.2023.1220835

**Published:** 2023-06-12

**Authors:** Jin-Wen Xu

**Affiliations:** Institute of Interdisciplinary Medical Science, Shanghai University of Traditional Chinese Medicine, Shanghai, China

**Keywords:** traditional Chinese medicine, cellular endocrinology, topics, prescription, active ingredient

Traditional Chinese medicine (TCM) is a long-established kind of alternative medicine that has been practiced in China for thousands of years. TCM is crucial for both the prevention and treatment of major diseases as well as for enhancing the health of the Chinese population. In recent years, TCM has been included in ICD-11, the 11th revision of the International Classification of Diseases, making it an integral part of global healthcare supply.

Experimental evidence suggests that the multi-target and multi-pathway integrated regulatory mode of action of TCM compound formulas may be beneficial for the treatment of a variety of diseases, including endocrine disorders. In addition to the use of experimental tools such as molecular biology and experimental pharmacology, TCM research has widely adopted various omics, network pharmacology, and chemical biology techniques to explore bioactive compounds, mechanisms of action, unidentified therapeutic targets, drug metabolism, and toxicological features.

In this Research Topic, several research reports and reviews on the treatment of endocrine-related diseases with herbal compound formulas and active ingredients are brought together. The topic covered in these studies include cellular endocrines in asthma and polycystic ovary syndrome, neuroendocrinology in Alzheimer’s disease, dyslipidemia in post-menopause, and inflammation and insulin resistance in nonalcoholic fatty liver disease. Fufang Zhenzhu Tiaozhi (FZT) Capsules and *Gynostemma Pentaphyllum* (GP) are compound formulas and single herbal medicines used in clinical practice. Their functions in the therapy of non-alcoholic steatohepatitis (NASH) have been clarified by two investigations (Lan et al.; Yue et al.). In a dose-dependent way, FTZ and GP polysaccharide alleviated the symptoms of obesity, insulin resistance, liver steatosis, liver fibrosis and oxidative stress in mice. FTZ attenuated the intestinal inflammatory response and improved intestinal barrier function, while GP polysaccharide inhibited Toll-like receptor 2 expression, down-regulated NLRP3 inflammasome activation, and decreased TNF-α and IL-1β levels (Lan et al.; Yue et al.). From a mechanistic perspective, changes in the composition of the gut microbiota play an essential role. According to gut microbiota sequencing, high-dose GP polysaccharides altered the composition of gut microbiota, significantly increasing the relative abundance of probiotic candidate strains (Yue et al.). Further, metabolomics analysis showed that compared to the high-fat feeding group, the FTZ group mice had higher overall levels of gut microbiota metabolites such as bile acids (Lan et al.). Menopausal women also have lipid metabolic and cardiovascular dysfunction issues, and their risk of hypercholesterolemia is significantly higher than that of premenopausal women. In addition to having low levels of circulating estradiol (E2), menopausal women also have a sharp increase in follicle stimulating hormone (FSH) levels during the course of the protracted menopause transition phase. Serum FSH levels are also positively correlated with total cholesterol levels. Elevated low-density lipoprotein cholesterol (LDL-C) in menopausal women is associated with a high risk of cardiovascular disease, and FSH may already reduce LDLR levels in hepatocytes, attenuating LDL-C endocytosis and resulting in raised levels of circulating LDL-C (1, 2). Tonifying Kidney and Removing Dampness Formula (TKRDF) is an herbal formula used clinically to improve dyslipidemia in postmenopausal women. Compared with the sham-operated group, the ovariectomized (OVX) rat model group exhibited the lowest serum E2 and the highest serum FSH. In comparison to the OVX group, serum E2 levels were significantly higher in the TKRDF and E2-treated groups, and serum FSH levels were significantly lower in the TKRDF-treated group compared to the model group (Li et al.). Using Protein-Protein Interaction network, Gene Ontology enrichment, Kyoto Encyclopedia of Genes and Genomes pathway analysis, and molecular docking methods, the authors found that TKRDF ameliorates postmenopausal dyslipidemia by regulating hormone levels, suppressing inflammation, promoting angiogenesis, and inhibiting lipid synthesis (Li et al.). Polycystic ovary syndrome (PCOS) is the most prevalent endocrine disorder in women of reproductive age and is the main cause of anovulatory subfertility. In PCOS, the hypothalamic release of gonadotropin-releasing hormone (GnRH) rises, prompting the pituitary gland to preferentially secrete luteinizing hormone (LH), resulting in ovarian hyperandrogenism and decreased ovulation. As a negative feedback, hyperandrogenemia reduces the response of estradiol to the hypothalamus, thereby maintaining a persistent rise in gonadotropin-releasing hormone (3, 4). Numerous formulas are believed to improve PCOS-related symptoms (Chen et al.). These formulas improve hyperandrogenism, reduce LH levels and LH/FSH ratios, enhance insulin sensitivity, elevate the number of follicles, promote the development of mature follicles, and increase ovulation rates (Chen et al.), indicating the enormous potential of Chinese herbal formulas in the treatment of PCOS. Unfortunately, none of these studies have observed changes in the hypothalamus. Only a few individual studies have found the inhibitory effect of herbal medicines on serum GnRH levels, such as the classic formul JinKui Sheqi pill (5) and the total flavonoids of some single herbs. Furthermore, Meng et al. reviewed and discussed the regulatory effects of various compound formulas and active ingredients on hormones, substances secreted by pulmonary neuroendocrine cells, and neuroendocrine related signaling proteins in the treatment of asthma. In addition, HPA axis mediated BDNF-regulated neuroplasticity has been linked to Alzheimer’s disease. Long-term endocrine abnormalities have direct negative consequences on the brain, such as neuronal metabolism, plasticity, and survival rate, resulting in cognitive deterioration. Clinical and experimental studies have confirmed that Chinese herbal medicine can improve the complex processes of these disorders, including metabolic abnormalities, neuroinflammation, and oxidative damage (Deng et al.).

TCM has been studied clinically and experimentally in the treatment of endocrine-related disorders, with some promising findings, but there are still numerous shortcomings. [1] most of the extracted herbal components cannot verify the purity of the compounds, lack quality control, and do not ensure the stability of the compounds; [2] most herbal studies are based on intragastric administration, and too little is known about drug metabolism; [3] the paucity of systematic studies on herbal medicines, and the unclear nature of the cellular and molecular mechanisms involved; [4] animal experiments do not take into account the toxic effects of herbal medicines. All of this implies that there is still a long way to go in interpreting and deciphering the enchantment of Chinese herbal medicine.

## Author contributions

The author confirms being the sole contributor of this work and has approved it for publication.
